# Polysiloxane-Based Polyurethanes with High Strength and Recyclability

**DOI:** 10.3390/ijms232012613

**Published:** 2022-10-20

**Authors:** Wencai Wang, Xueyang Bai, Siao Sun, Yangyang Gao, Fanzhu Li, Shikai Hu

**Affiliations:** 1 State Key Laboratory of Organic-Inorganic Composites, Beijing University of Chemical Technology, Beijing 100029, China; 2 Key Laboratory of Carbon Fiber and Functional Polymers, Ministry of Education, Beijing University of Chemical Technology, Beijing 100029, China; 3 Beijing Engineering Research Center of Advanced Elastomers, Beijing University of Chemical Technology, Beijing 100029, China

**Keywords:** polysiloxane-based, polyurethane, high strength, recyclability, microphase separation

## Abstract

Polysiloxanes have attracted considerable attention in biomedical engineering, owing to their inherent properties, including good flexibility and biocompatibility. However, their low mechanical strength limits their application scope. In this study, we synthesized a polysiloxane-based polyurethane by chemical copolymerization. A series of thermoplastic polysiloxane-polyurethanes (Si-TPUs) was synthesized using hydroxyl-terminated polydimethylsiloxane containing two carbamate groups at the tail of the polymer chains 4,4′-dicyclohexylmethane diisocyanate (HMDI) and 1,4-butanediol as raw materials. The effects of the hard-segment content and soft-segment number average molecular weight on the properties of the resulting TPUs were investigated. The prepared HMDI-based Si-TPUs exhibited good microphase separation, excellent mechanical properties, and acceptable repeatable processability. The tensile strength of SiTPU-2K-39 reached 21.5 MPa, which is significantly higher than that of other flexible polysiloxane materials. Moreover, the tensile strength and breaking elongation of SiTPU-2K-39 were maintained at 80.9% and 94.6%, respectively, after three cycles of regeneration. The Si-TPUs prepared in this work may potentially be used in gas separation, medical materials, antifouling coatings, and other applications.

## 1. Introduction

Polysiloxanes possess a macromolecular backbone composed of repeating Si–O–Si bonds, with the direct attachment of organic groups to Si atoms. These materials are characterized by the excellent properties of both inorganic and organic materials [1,2,3,4]. The inorganic properties of Si materials effectively improve their heat resistance and flame retardancy [5], while their high Si–O–Si bond energy and low surface energy endow them with outstanding wear and weathering resistance [6]. In addition, polysiloxanes exhibit good hydrophobicity [7,8], high flexibility [9], and biocompatibility [10]. However, their low mechanical strength limits their applications in various fields [11,12].

The mechanical properties of polysiloxanes can be effectively improved by modification with other polymeric materials, such as epoxy resin [13], polyurea [14], polyether [15], and polyurethane [16]. Modification with polyurethane presents great significance as a modification technique because the molecule has a well-regulated structure. Polyurethanes are composed of alternating soft segments (SS) and hard segments (HS); SS confers flexibility and toughness, whereas HS provides rigidity, strength, wear resistance, and excellent mechanical properties [17,18,19,20]. Current methods to modify polysiloxanes using polyurethanes mainly include physical blending [21] and chemical copolymerization [22]. However, the solubility parameters of polyurethane and polysiloxane remarkably differ because of their different molecular structures; thus, these materials usually show poor compatibility [23]. Modification by simple physical blending can lead to high interfacial tension and poor interfacial adhesion [24], resulting in macroscopic phase separation. In this case, polysiloxane easily migrates toward the surface of the blended material [25,26] because no chemical bonds are generated between polysiloxane and polyurethane. Thus, the stability of the modified Si polyurethane is poor, and the aging properties of the material are reduced. Modification by physical blending cannot meet the needs of most actual applications. Chemical modification involves the use of end-functionalized polysiloxanes for copolymerization with organic polymers to obtain new polymeric materials [27,28,29]. This technique fundamentally improves material compatibility and solves the drawbacks of physical blending [30,31,32].

Conventional polyurethane raw materials, such as polyether and polyester polyols, are petroleum-dependent products, which considerably limit their sustainable development [33,34,35,36]. The Earth’s crust contains over 90 elements, with O and Si accounting for approximately 49% and 26% of the total abundance of these elements, respectively [37]. The utilization of Si resources can reduce energy consumption, improve the atmospheric environment, and realize sustainable economic development. Therefore, polysiloxane-based polyurethane materials have great development and application prospects. These modified materials can overcome the poor mechanical properties of polysiloxanes, rendering them applicable to textiles [38], medical materials [39,40,41], adhesives [42], and coatings [43,44,45], among others. Compared with aromatic isocyanates [46], aliphatic 4,4-dicyclohexylmethane diisocyanate (HMDI) and isophorone diisocyanate (IPDI) are ideal materials for synthesizing aliphatic polyurethanes for biomedical applications, owing to their nontoxicity and yellowing resistance [47,48,49]. Moreover, the water and heat resistances of HMDI-based TPU are better than those of IPDI-based TPUs. Therefore, in this study, HMDI was selected as the HS for synthesizing polyurethane.

In this work, hydroxyl-terminated polydimethylsiloxane (PDMS; HO–PDMS–OH) was selected to synthesize polysiloxane-based polyurethanes because amino-terminated PDMS (H_2_N–PDMS–NH_2_) reacts violently with isocyanate groups. However, HO–PDMS–OH is expensive, and the necessary technology is relatively immature when compared with the H_2_N–PDMS–NH_2_ approach; therefore, HO–PDMS–OH was presynthesized by reacting H_2_N–PDMS–NH_2_ and vinyl carbonate (EC), as described in our previous work [50]. Compared to conventional hydroxy silicone oil, the prepared OH–PDMS–OH possessed higher polarity and better compatibility with diisocyanates because of the l urethane groups. In addition, it could also increase hydrogen bond interactions, hence, improving mechanical properties [51]. Next, a series of polysiloxane-polyurethanes (Si-TPUs) was prepared by reacting HO–PDMS–OH, 4,4-dicyclohexylmethane diisocyanate (HMDI), and the chain extender 1,4-butanediol (BDO). The effects of SS number average molecular weight (*M_n_*) and HS content on the microstructure and properties of the resultant Si-TPUs were evaluated. Overall, the prepared Si-TPUs exhibited good phase separation, excellent mechanical strength, and acceptable repeatable processability. Therefore, chemical copolymerization is a highly effective method for modifying polysiloxanes and can broaden their application areas.

## 2. Experimental Section

### 2.1. Materials

Aminopropyl-terminated PDMS (H_2_N–PDMS–NH_2_, *M_n_* = 1000, 2000, and 3000) was purchased from Beijing Warwick Chemical Co. Ltd. (Beijing, China). EC was obtained from Shanghai Adamas Reagent Co., Ltd. (Shanghai, China). Analytical-reagent-grade HMDI was obtained from Shanghai Macklin Biochemical Technology Co., Ltd. (Shanghai, China). Analytical-reagent-grade BDO was supplied by Shanghai Aladdin Biochemical Technology Co., Ltd. (Shanghai, China). Analytical-reagent-grade tetrahydrofuran (THF) was obtained from Beijing Chemical Works. Analytical-reagent-grade dibutyltin dilaurate (DBTL) was supplied by Hebei Bailing Weichao Fine Materials Co., Ltd. (Hebei, China).

### 2.2. Synthesis of the HMDI-Based Thermoplastic Polyurethanes

**Scheme 1:**
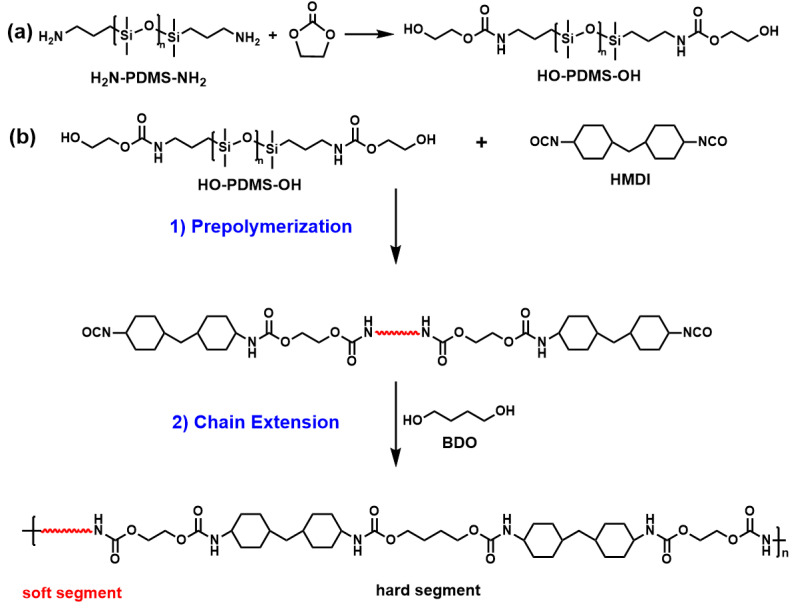
(**a**) Synthetic route of HO–PDMS–OH; (**b**) synthetic route of HMDI-based Si-TPUs.

The SS HO–PDMS–OH was first prepared according to a previously described method [50]. Next, the HMDI-based Si-TPU was synthesized as follows. An oil bath was heated to 115 °C. A measured amount of HO–PDMS–OH (20 mmol) was then added to a three-necked flask equipped with a mechanical stirrer, nitrogen inlet, and condensate tube for 1.5 h of vacuum distillation. The temperature was reduced to 75 °C, and a measured amount of HMDI and two drops of the catalyst DBTL were added to the flask. The temperature was increased to 85 °C for prepolymerization and held for 2 h. Next, a measured amount of the chain extender BDO was introduced to the flask at 90 °C and mixed for 0.5 h. Finally, the obtained HMDI-based Si-TPU was poured into tetrafluoroethylene molds and cured for 12 h at 100 °C. The prepared Si-TPUs were denoted SiTPU-X-Y, where X represents the *M_n_* value of SS and Y represents the HS content. The synthetic procedure is shown in Scheme 1, and the ratios of the raw materials are shown in Table 1.

### 2.3. Characterization

#### 2.3.1. FT-IR Spectroscopy

FT-IR spectroscopy was performed on a Tensor 27 FT-IR spectrometer (Bruker, Bremen, Germany) under the following test conditions: test mode = ATR, wavenumber range = 500–4000 cm^−1^, resolution = 4 cm^−1^, and scanning number = 32.

#### 2.3.2. Gel Permeation Chromatography (GPC)

GPC was performed using a Shimadzu GPC chromatograph (Kyoto, Japan). The Si-TPU samples were dissolved in THF and prepared into a solution of 10 mg/mL. The flow velocity was 1 mL/min. Polystyrene was used as the calibration standard.

#### 2.3.3. Differential Scanning Calorimetry (DSC)

The DSC thermograms of the samples were measured on an STARe System DSC instrument (Mettler-Toledo, Greifensee, Switzerland). First, the temperature was reduced from 30 °C to −140 °C at a rate of −10 °C/min, and then maintained at this temperature for 10 min. The temperature was then increased to 100 °C at a rate of 10 °C/min under a continuous N_2_ purge.

#### 2.3.4. Wide-Angle X-ray Diffraction (WAXD)

WAXD spectra were recorded using a D/Max 2500 VB2+/PC model (Rigaku Corporation, Tokyo, Japan). The Si-TPU samples were prepared as flat flakes with a thickness of 1 mm at room temperature. Measurements were performed at a scanning rate of 5 °C/min in the 2θ range of 5–90°.

#### 2.3.5. Small-Angle X-ray Scattering (SAXS)

SAXS measurements were performed on a Xuess2-0 scatterer (Xenocs, Grenoble, France) at a wavelength of 0.154 nm. Herein, 2D SAXS patterns were acquired using a Pilatus 200 K detector. The distance between the detector and all samples, except SiTPU-1K-33, was 2490 mm; SiTPU-1K-33 was positioned at 1670 mm from the detector. The exposure time was set to 20 min.

#### 2.3.6. Atomic Force Microscopy (AFM)

AFM measurements were performed using a Multimode 8 instrument (Bruker, Bremen, Germany) in tapping mode, with a scan area of 2 × 2 μm^2^. The sample surface was polished well to enable precise measurements.

#### 2.3.7. Tensile Testing

Tensile testing was performed using a Roell Txet-port II instrument (Zwick, Ulm, Germany) at room temperature. The strain rate was set to 50 mm/min. The mechanical properties of the Si-TPU products were tested using dumbbell-type flake splines with a thickness, width, and length of 1, 4, and 25 mm, respectively.

#### 2.3.8. Repeatable Processability

The used samples were sheared into small pieces and molded into 1 mm films by a vulcanizer for reprocessing. The test conditions used to assess the repeatable processability of the samples were identical to those used to test their mechanical properties.

## 3. Results and Discussion

### 3.1. FT-IR Spectral Analysis

**Figure 1:**
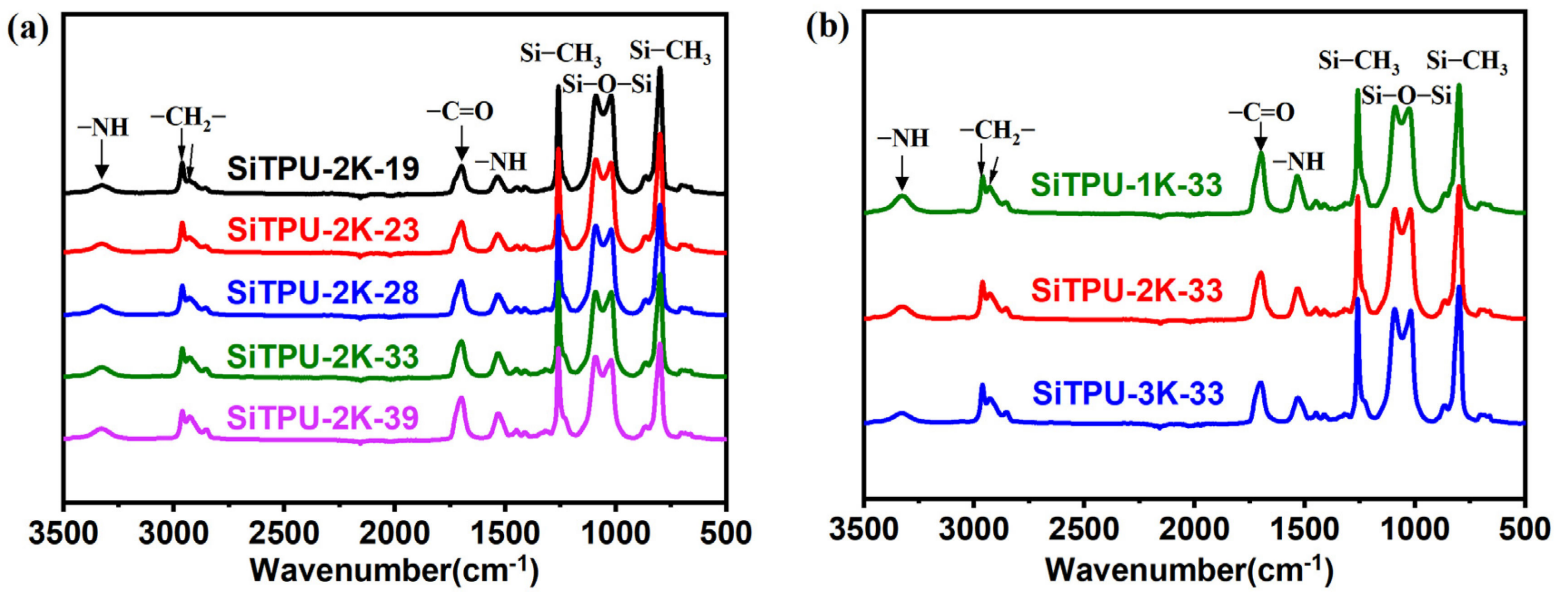
(**a**) FT-IR spectra of the Si-TPUs with different HS contents. (**b**) FT-IR spectra of the Si-TPUs with different *M_n_* values of the SS.

The chemical structures of the synthesized Si-TPUs were analyzed using FT-IR, as shown in Figure 1. The characteristic absorption peak of the NCO group in HMDI, which occurs at 2264 cm^−1^, disappeared [52], indicating the complete reaction of this group. The characteristic peaks at 1708–1741 and 3322–3412 cm^−1^ correspond to the stretching vibrations of −C=O and −NH, respectively. The stretching and asymmetric bending vibrations of Si–CH_3_ were observed at 1255–1261 and 791–812 cm^−1^, respectively. The “M”-type peaks located at 1094–1097 and 1025–1028 cm^−1^ are the characteristic absorption peaks of Si–O–Si. The peaks at 2961–2972 and 2932–2937 cm^−1^ represent the asymmetric and symmetric stretching vibrations of –CH_2_, respectively [15,21]. Thus, the FT-IR results confirmed the successful synthesis of the HMDI-based Si-TPUs.

The peaks in the carbonyl region from 1610 to 1800 cm^−1^ could be resolved into two peaks at 1699 and 1726 cm^−1^ (Figures S1 and S2), corresponding to hydrogen-bonded and free carbonyl groups, respectively [53]. Hydrogen bonding associations (HBA) are related to the proportion of carbonyl groups and can be calculated using the following formula [54]:

$$HBA=\frac{S_{1699}}{S_{1699}+S_{1726}}\times 100\%,$$

where *S*_1699_ represents the area of the hydrogen-bonded carbonyl peak at 1699 cm^−1^ and *S*_1726_ represents the area of the free carbonyl peak at 1726 cm^−1^. Table 2 lists the percentages of hydrogen-bonded and free carbonyl groups of the synthesized materials. As the HS content of the Si-TPUs increased, the proportion of hydrogen-bonded carbonyls increased and the proportion of free carbonyls decreased. These changes could be explained by the increase in number of hydrogen bonds formed between HS and separation of more HS from SS with increasing HS content. In addition, the proportion of hydrogen-bonded carbonyls decreased, whereas the proportion of free carbonyls increased as the SS *M_n_* increased, possibly because of the increase in distance between carbamate groups, which decreases their density. Variable-temperature IR spectroscopy (Figure S3) indicated the dissociation of hydrogen bonds due to elevations in temperature. The proportion of hydrogen-bonded carbonyls and amino groups decreased, whereas that of free carbonyls and amino groups increased when the temperature was increased from 30 to 180 °C.

### 3.2. GPC

**Figure 2:**
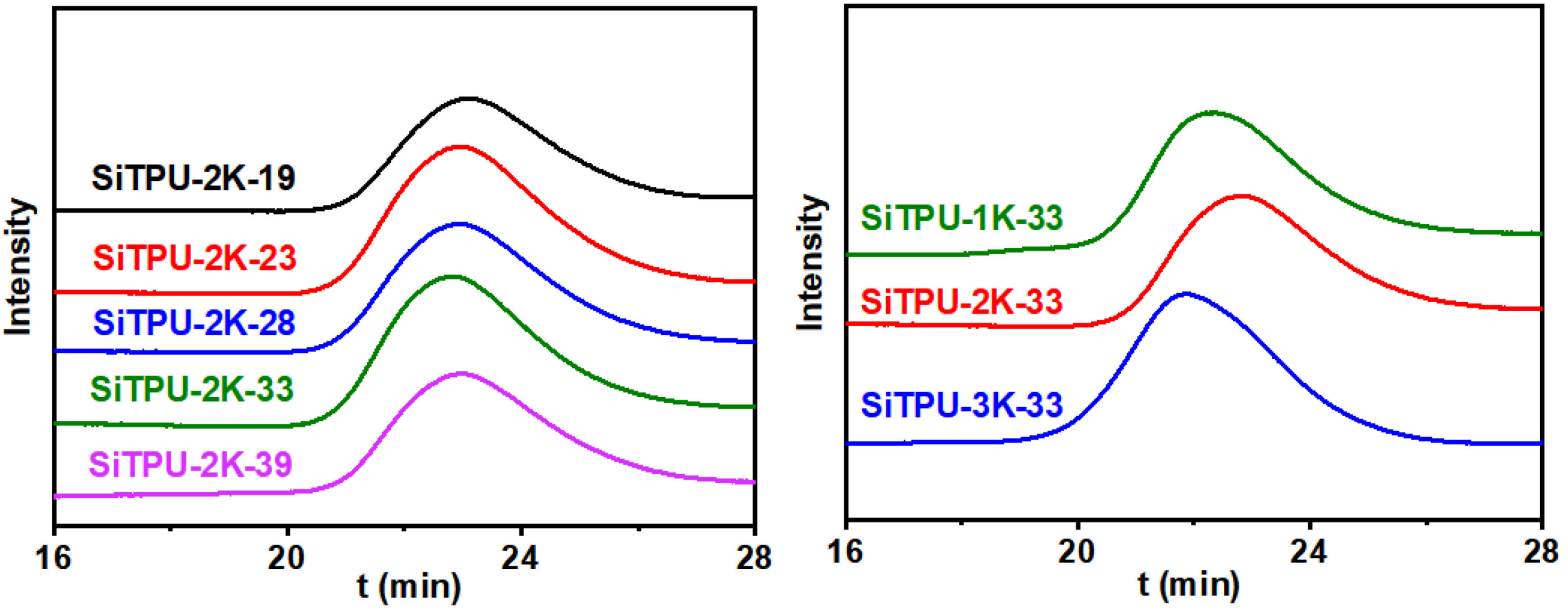
GPC curves of the HMDI-based Si-TPUs.

Figure 2 shows the GPC curves of the synthesized Si-TPUs, and Table S1 summarizes their molecular weights and molecular weight distributions (Ð). The Si-TPU samples were dissolved in THF. The solutions completely passed through the filter membrane without insoluble particles, thus confirming the uncross-linked structure and high solubility of the synthesized HMDI-based Si-TPUs. The GPC results confirmed the relatively close values of *M_n_* and average molecular weight (*M_w_*), and all values obtained were greater than 25,000. The Ð values of all compounds were approximately 1.9.

### 3.3. DSC

**Figure 3:**
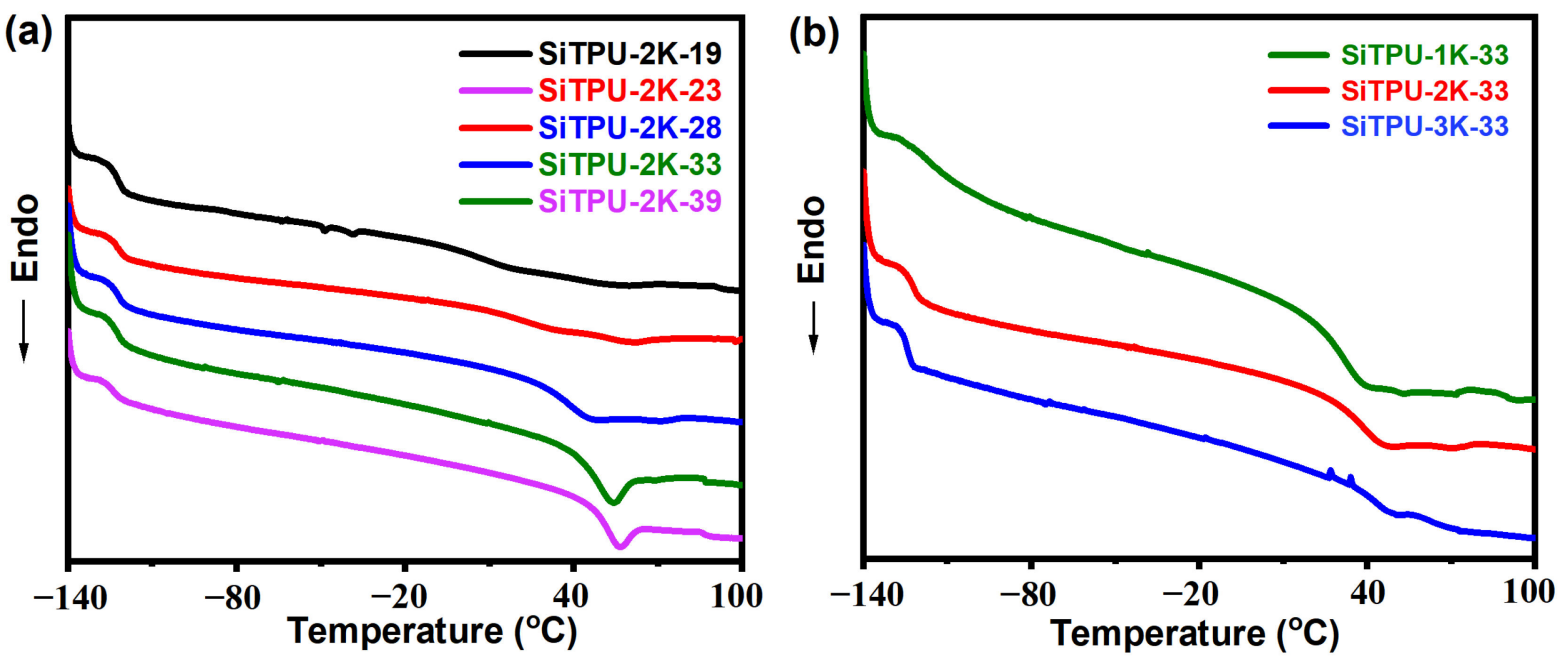
(**a**) DSC curves of the Si-TPUs with different HS contents (**b**) DSC curves of the Si-TPUs with different *M_n_* values of the SS.

The DSC curves of Si-TPUs with different HS contents and SS *M_n_* values are shown in Figure 3a,b, respectively. The Si-TPUs had two glass transition temperatures (*T_g_*). *T_g_*_1_, which is related to the SS and appeared at −123 °C, was barely influenced by the HS content and SS *M_n_* value, whereas *T_g_*_2_, which appeared at approximately 40 °C, was related to HS. As shown in Figure 3a, when the SS *M_n_* was held constant, *T_g_*_2_ gradually increased as the HS content increased, mainly because the number of hydrogen bonds between HS increases with increasing HS content. However, the mobility of HS is limited. Thus, the energy required to move these segments increases, leading to a higher *T_g_*_2_. As shown in Figure 3b, *T_g_*_2_ gradually increased with increasing SS *M_n_*, mainly because the average HS *M_n_* also increases as the SS *M_n_* increases when the HS content is held constant. Increases in the number of hydrogen bonds between HS lead to a higher *T_g_*_2_.

### 3.4. WAXD

**Figure 4:**
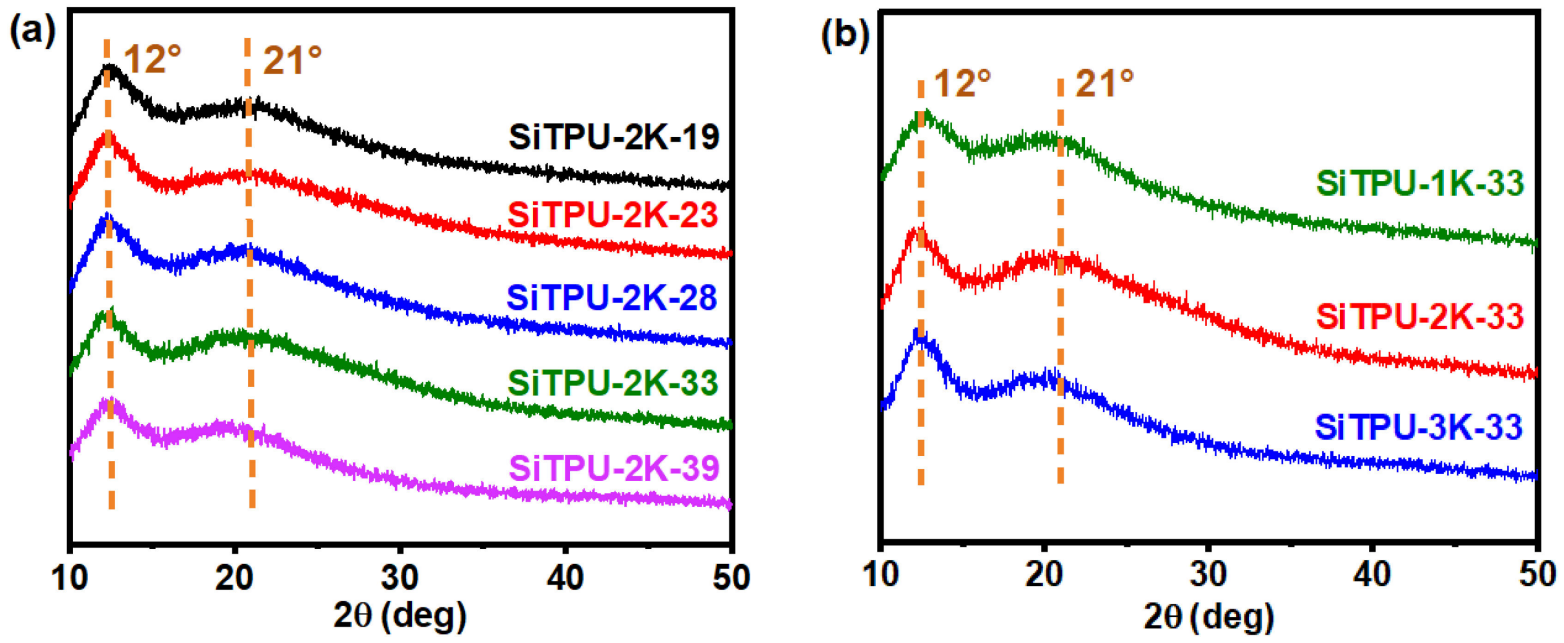
(**a**) WAXD curves of the Si-TPUs with different HS contents. (**b**) WAXD curves of the Si-TPUs with different *M_n_* values of the SS.

Figure 4a,b show the 1D-WAXD curves of the prepared Si-TPUs. The broad diffusion peak at 2θ = 12° was assigned to the amorphous phase of PDMS, whereas that at 21° was assigned to the non-PDMS segments of the polymer chains [55,56]. Therefore, the SS and HS regions in the HMDI-based Si-TPUs similarly exhibited an amorphous morphology without sharp peaks.

### 3.5. SAXS

**Figure 5:**
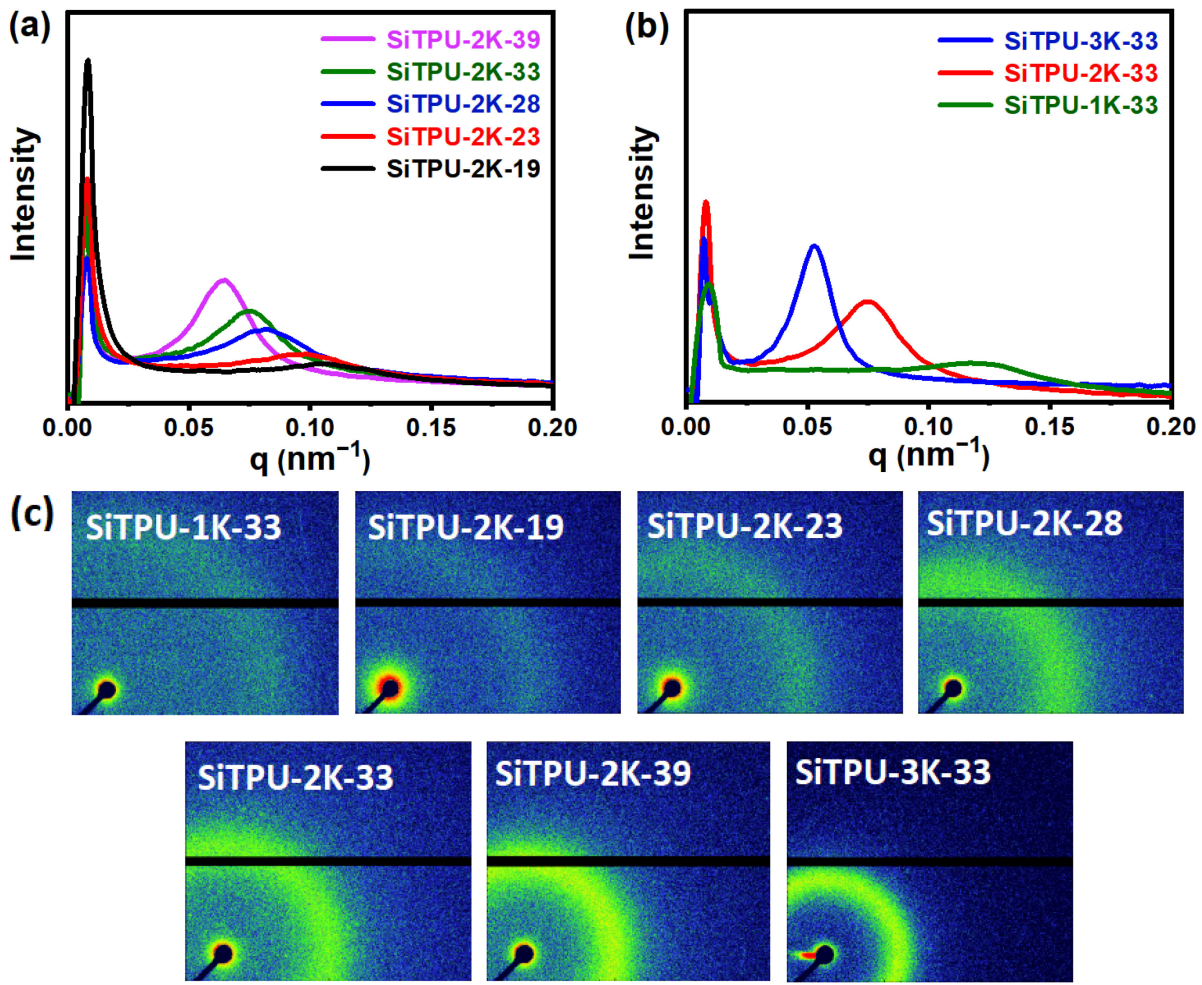
(**a**) 1D SAXS curves of Si-TPUs with different HS contents. (**b**) 1D SAXS curves of SiTPU-1K-33, SiTPU-2K-33, and SiTPU-3K-33. (**c**) 2D SAXS patterns of the HMDI-based Si-TPUs.

Figure 5a,b show the 1D-SAXS curves of the HMDI-based Si-TPUs with different HS contents and SS *M_n_* values, respectively. Figure 5c shows the 2D-SAXS patterns of the Si-TPUs. The periodic sizes (D) of the synthesized materials are presented in Table S2. D reflects the mean distance between SS and HS regions and was calculated using the formula D = 2π/*q*_max_ [57,58], where *q*_max_ represents the *q* value of the maximum scattering peak. The peaks and isotropic scattering rings shown in Figure 5 confirm that the HMDI-based Si-TPUs have microphase separation structures. Moreover, the scattering peak was gradually enhanced, the scattering loop became clearer, and D value increased as the HS content increased. These findings can be attributed to increases in HS average molecular weight and degree of phase separation with increasing HS content. Figure 5 and Table S2 show that *q*_max_ gradually decreases, whereas D increases, as the SS *M_n_* increases. In addition, the scattering peaks and loops are enhanced, mainly because the size of the SS microdomain and average HS *M_n_* increase as the SS *M_n_* increases. Thus, D and the degree of microphase separation increase accordingly, rendering the scattering peaks and rings more evident.

### 3.6. AFM

**Figure 6:**
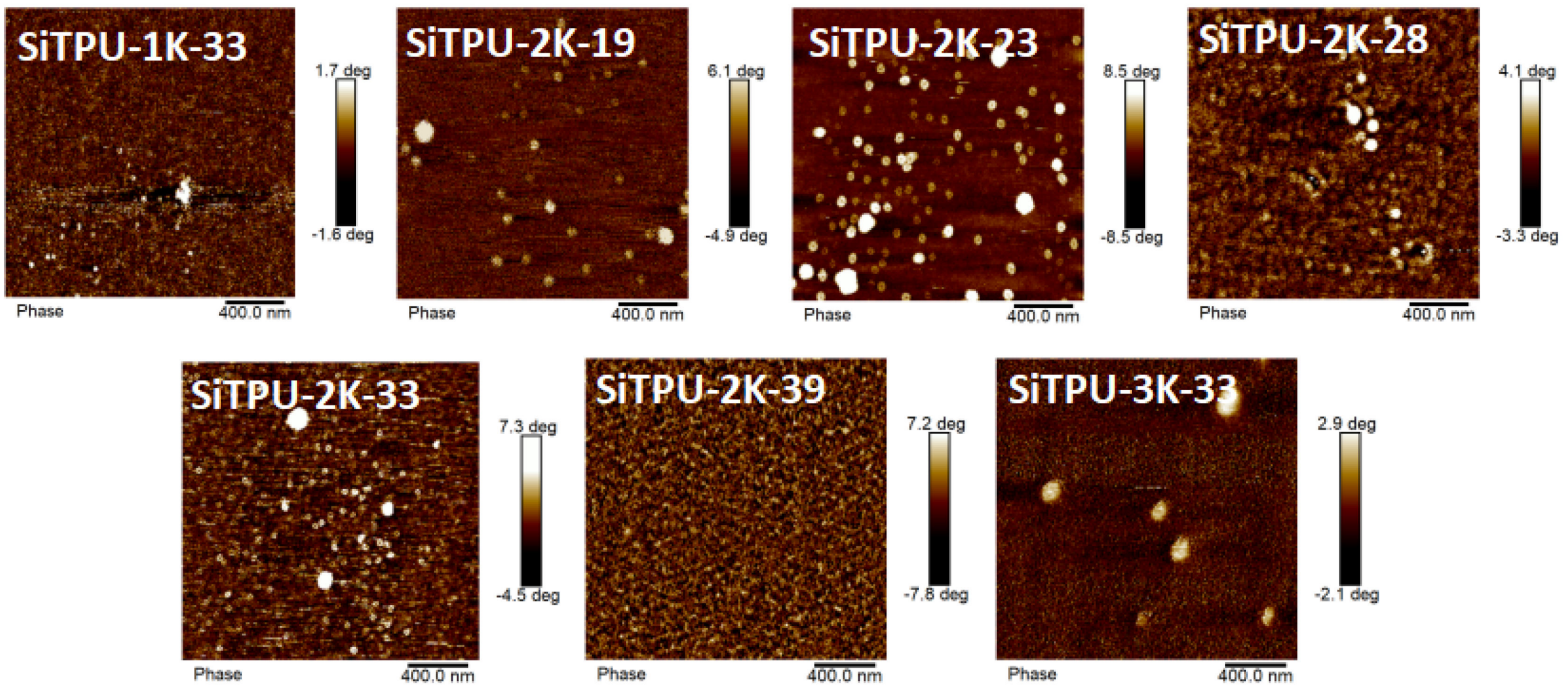
AFM images of the HMDI-based Si-TPUs.

The microstructure of the Si-TPUs was investigated using tapping-mode AFM, as shown in Figure 6, to clarify their microphase separation structures. In the images obtained, the bright areas represent HS domains, whereas the dark areas represent SS domains. The AFM images reveal that the HS domains are evenly distributed in the SS phase and that the HMDI-based Si-TPUs exhibit excellent phase separation, which is consistent with the SAXS results. The AFM image of SiTPU-2K-39 reveals that the HS forms a continuous phase; moreover, the SS and HS domains are evenly distributed. By comparison, fewer HS domains are visible in the field of view of SiTPU-3K-33. The longer SS domains of SiTPU-3K-33 compared with those of SiTPU-1K-33 and SiTPU-2K-33 likely caused a reduction in the density of HS domains.

### 3.7. Mechanical Properties

**Figure 7:**
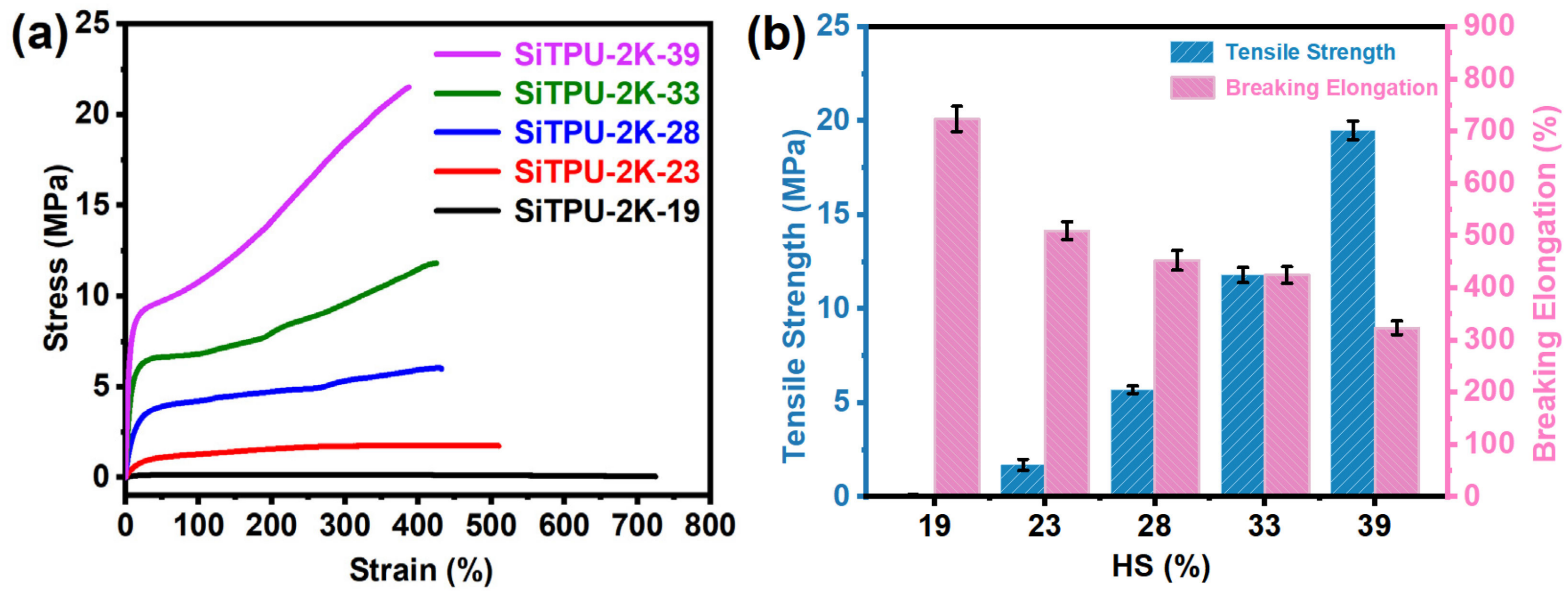

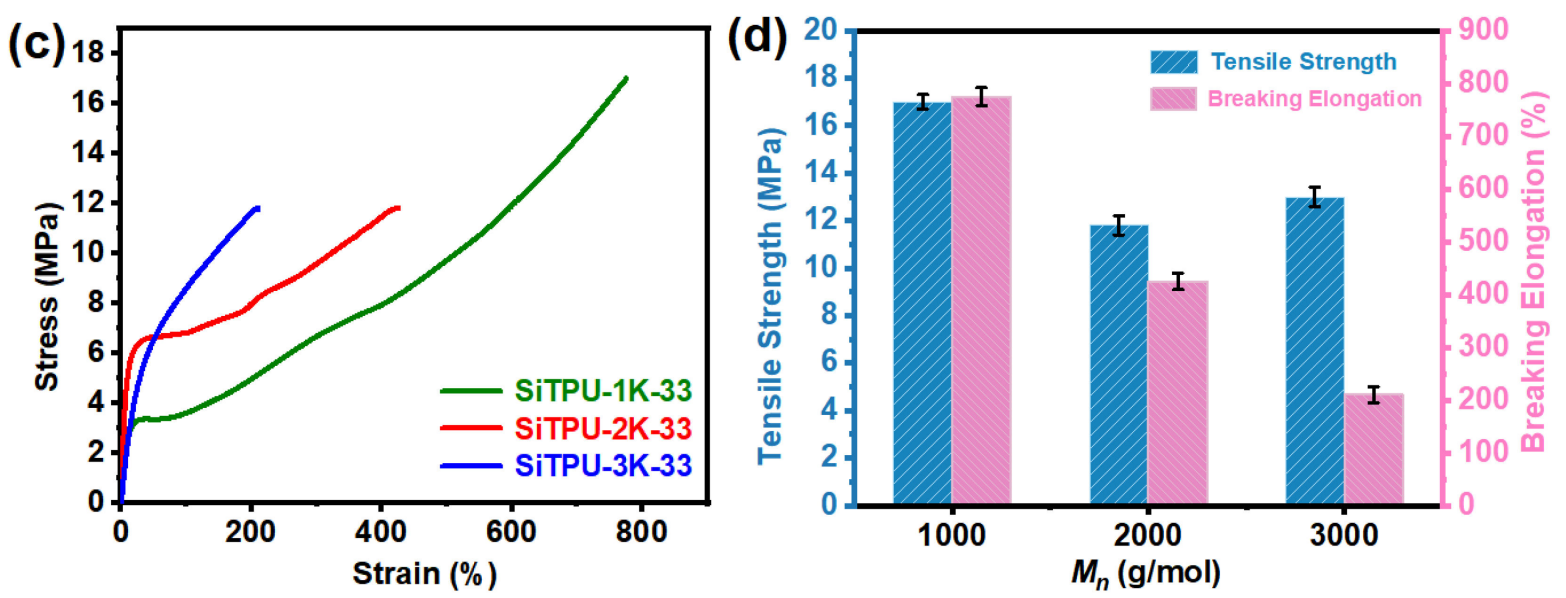
(**a**) Stress−strain curves of the Si-TPUs with different HS contents. (**b**) A histogram of mechanical properties of Si-TPUs with different HS contents. (**c**) Stress−strain curves of the Si-TPUs with different *M_n_* values of the SS. (**d**) A histogram of mechanical properties of Si-TPUs with different *M_n_* values of the SS.

Curves of the various mechanical properties of the HMDI-based Si-TPUs are shown in Figure 7, and the detailed mechanical property data are listed in Table S3. As shown in Figure 7a,b, when the SS *M_n_* was held constant, the tensile strength of the Si-TPUs increased significantly, whereas their breaking elongation decreased as the HS content increased. This finding may be attributed to the increase in number of hydrogen bonds and physical crosslinking points between polymer chains when the HS content is increased. Thus, the tensile strength of the Si-TPUs increased. However, excess physical crosslinking points limit the slippage of polymer chains; therefore, the breaking elongation of the samples gradually decreased. As shown in Figure 7c,d, when the HS content was held constant, the elongation at break of the Si-TPUs decreased as the SS *M_n_* increased, likely because the average length of the HS domains increases as the SS *M_n_* increases. The number of hydrogen bonds also increases, thereby restricting the deformation of the materials. As the degree of physical crosslinking increases, the stress required to deform the materials uniformly increases; thus, their tensile stress at 100% breaking elongation increases. The ultimate tensile strength of SiTPU-1K-33 reached 17 MPa; such high strength could be attributed to the molecular orientation in the stretching direction and strain-induced crystallization during the stretching process [59,60]. These results collectively suggest that the HMDI-based Si-TPUs possess excellent mechanical properties.

### 3.8. Repeatable Processability

**Figure 8:**
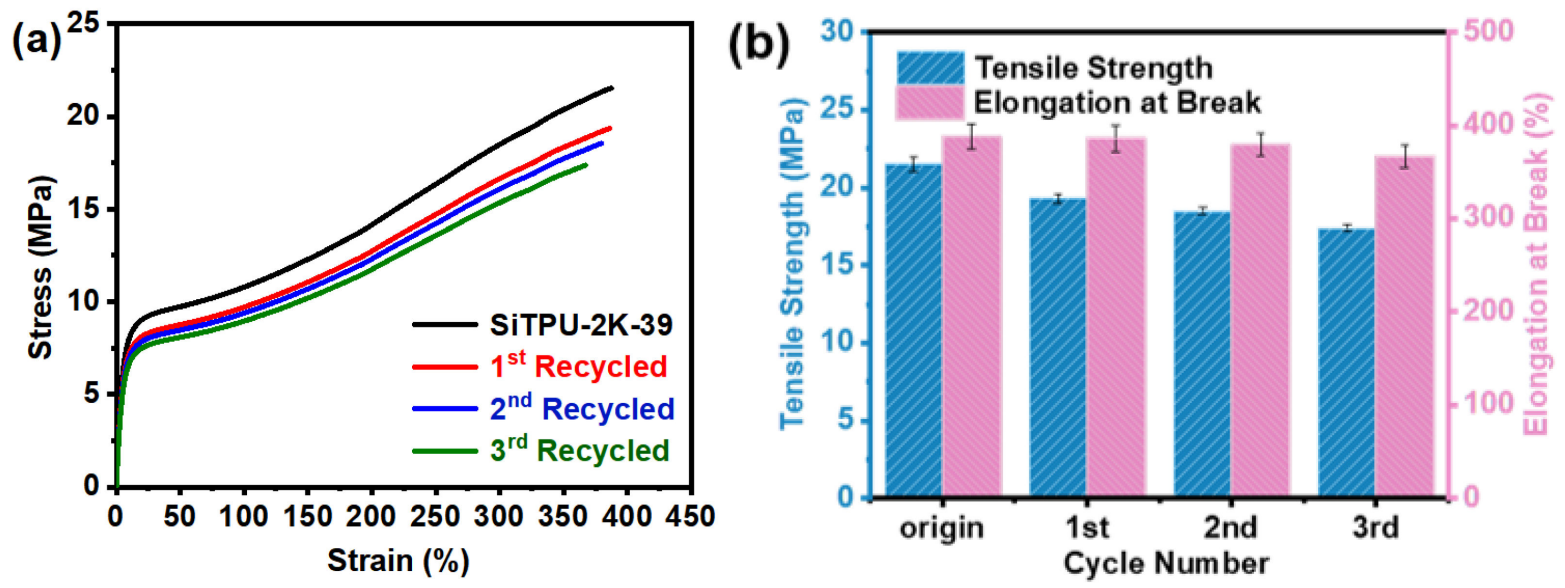
(**a**) Representative stress−strain curves of PDMS2K-39. (**b**) A histogram of mechanical properties of PDMS2K-39.

SiTPU-2K-39 had the highest tensile strength among the prepared Si-TPUs. Figure 8 presents its mechanical properties after three cycles of processing; the corresponding data are presented in Table S4. As shown in Figure 8, after three cycles of processing, the mechanical properties of SiTPU-2K-39 slightly decreased. The tensile strength of the sample decreased from 21.5 to 17.4 MPa, with a retention rate of 80.9%, and its elongation at break decreased from 388% to 367%, with a retention rate of 94.6%. The tensile stresses at 100% and 300% elongations were also slightly reduced. These changes may be mainly attributed to the generation of small molecular substances, which affect the mechanical properties of the sample, during repetitive processing. However, the sample maintained at least 80% of its original mechanical strength, indicating that the HMDI-based Si-TPUs have good repeatable processability.

## 4. Conclusions

In this study, we successfully prepared thermoplastic HMDI-based Si-TPUs via a two-step reaction using HO–PDMS–OH, HMDI, and BDO. The Si-TPUs exhibited microphase separation, which influenced their mechanical properties, owing to the incompatibility between SS and HS. As the HS content increased, the tensile strength of the TPUs increased, whereas their breaking elongation decreased. When the HS content was held constant, the breaking elongation of the TPUs decreased with increasing SS *M*_n_. The tensile strength of SiTPU-1K-33 reached 17.0 MPa. The mechanical properties of SiTPU-2K-39 were slightly degraded after three cycles of reprocessing. The tensile strength and breaking elongation of the sample were well maintained, with retention rates of 80.9% and 94.6%, respectively. These results demonstrate the good reusability and clear potential of the synthesized Si-TPUs for developing recycled materials. Overall, the block introduction of polysiloxanes into the polyurethane system by copolymerization can compensate for the individual deficiencies of these materials and enhance their comprehensive properties. Thus, Si-TPU-block copolymers are promising polymeric materials that can be widely utilized in various applications, such as biomedical materials, elastomers, functional coatings, and sealants.

## Figures and Tables

**Table 1:** Chemical composition of the synthesized HMDI-based Si-TPUs.

Sample	SS *M_n_*	HS Content	PDMS:HMDI:BDO (Molar Ratio)
SiTPU-1K-33	1000	33%	1:1.15:0.15
SiTPU-2K-19	2000	19%	1:1.1:0.1
SiTPU-2K-23	2000	23%	1:1.45:0.45
SiTPU-2K-28	2000	28%	1:2:1
SiTPU-2K-33	2000	33%	1:2.5:1.5
SiTPU-2K-39	2000	39%	1:3.4:2.4
SiTPU-3K-33	3000	33%	1:4:3

**Table 2:** Percentage of free and hydrogen-bonded carbonyl groups obtained from fitted-curve analysis.

Sample	Hydrogen-Bonded Carbonyls (1699 cm^−1^)	Free Carbonyls (1726 cm^−1^)
SiTPU-1K-33	81.37	18.63
SiTPU-2K-19	77.30	22.70
SiTPU-2K-23	77.96	22.04
SiTPU-2K-28	78.50	21.50
SiTPU-2K-33	80.85	19.15
SiTPU-2K-39	83.82	16.18
SiTPU-3K-33	79.21	20.79

## Data Availability

Not applicable.

## Supplementary Materials

The following supporting information can be downloaded at: https://www.mdpi.com/article/10.3390/ijms232012613/s1. Figure S1. (a) FTIR spectra of Si-TPUs in the carbonyl regions from 1610 to 1800 cm^−1^. Fitted Curve of the carbonyl groups of SiTPU-2K-19 (b), SiTPU-2K-23 (c), SiTPU-2K-28 (d), SiTPU-2K-33 (e) and SiTPU-2K-39 (f). Figure S2. (a) FTIR spectra of Si-TPUs in the carbonyl regions from 1610 to 1800 cm^−1^. Fitted Curve of the carbonyl groups of SiTPU-1K-33 (b), SiTPU-2K-33 (c), SiTPU-3K-33 (d). Figure S3. FTIR spectra of (a) N–H and (b) C=O from SiTPU-2K-39. Table S1. Molecular weights and molecular weight distribution indices of Si-TPUs. Table S2. Periodic size (D) of the Si-TPUs. Table S3. Summary of the mechanical performance values of Si-TPUs. Table S4. The mechanical performance values of Si-TPUs after multiple recycles.
