# Supplementary material for: Polysiloxane-Based Polyurethanes with High Strength and Recyclability
Source: Int J Mol Sci. 2022 Oct 20;23(20):12613. doi: 10.3390/ijms232012613 (PMC9604122; doi:10.3390/ijms232012613)
Supplement: Supplementary file 1 [file ijms-23-12613-s001.zip › ijms-1944558-supplementary.pdf]

Supporting Information

Polysiloxane-Based Polyurethanes with High-Strength and Recyclability

Wencai Wang[1,2,3], Xueyang Bai[1], Siao Sun[1], Yangyang Gao[1,2,3], Fanzhu Li[1,2,3], Shikai Hu[1,2,3*]*

[1] State Key Laboratory of Organic-Inorganic Composites, Beijing University of Chemical Technology, Beijing 100029, China

[2] Key Laboratory of Carbon Fiber and Functional Polymers, Ministry of Education, Beijing University of Chemical Technology, Beijing 10029, China

[3] Beijing Engineering Research Center of Advanced Elastomers, Beijing University of Chemical Technology, Beijing 100029, China.

* Corresponding author:

lifz@mail.buct.edu.cn (Fanzhu Li);

skhu@mail.buct.edu.cn (Shikai Hu)

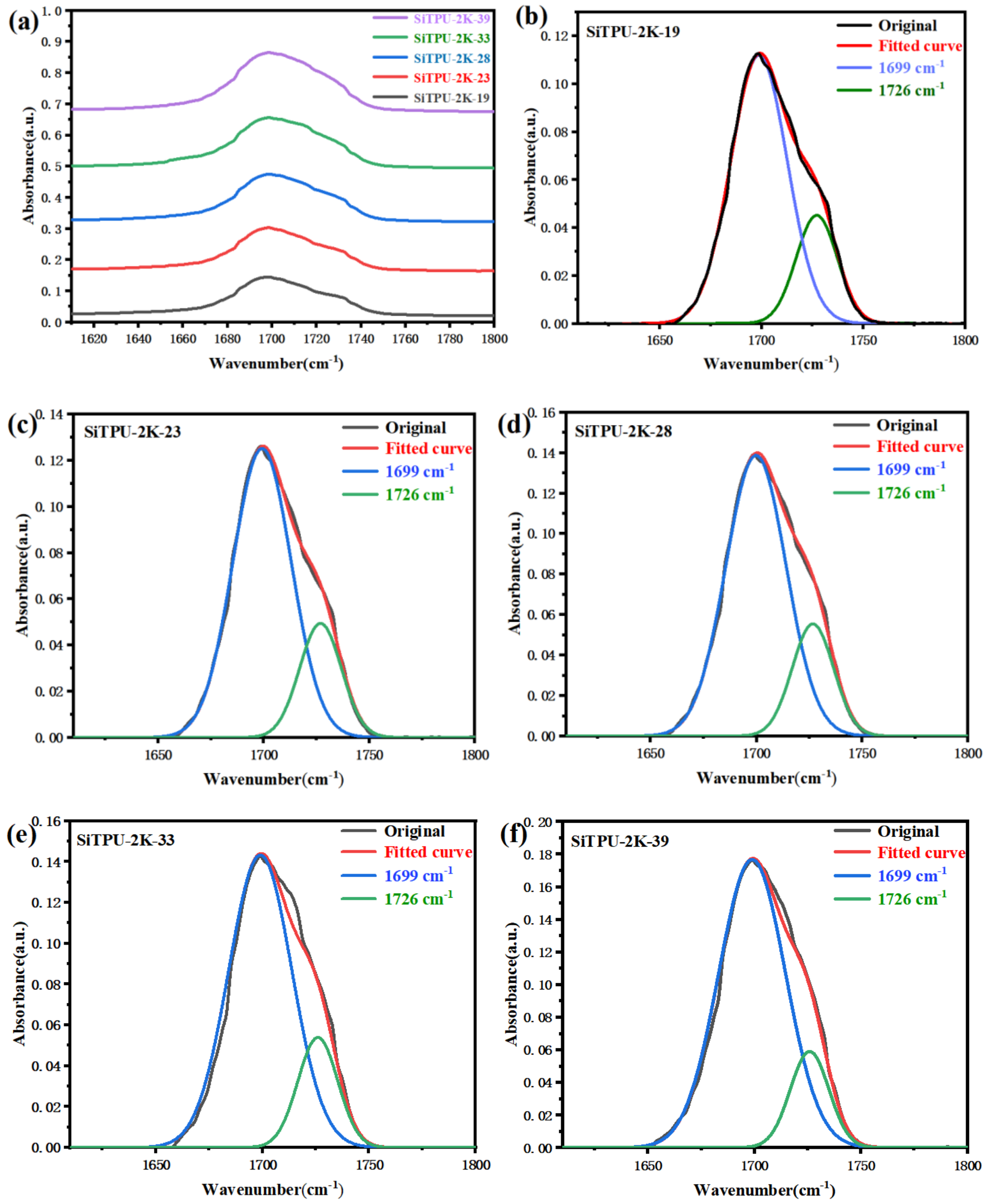

Fig S1. (a) FTIR spectra of Si-TPUs in the carbonyl regions from 1610 to 1800 cm^{-1}. Fitted Curve of the carbonyl groups of SiTPU-2K-19 (b), SiTPU-2K-23 (c), SiTPU-2K-28 (d), SiTPU-2K-33 (e) and SiTPU-2K-39 (f).

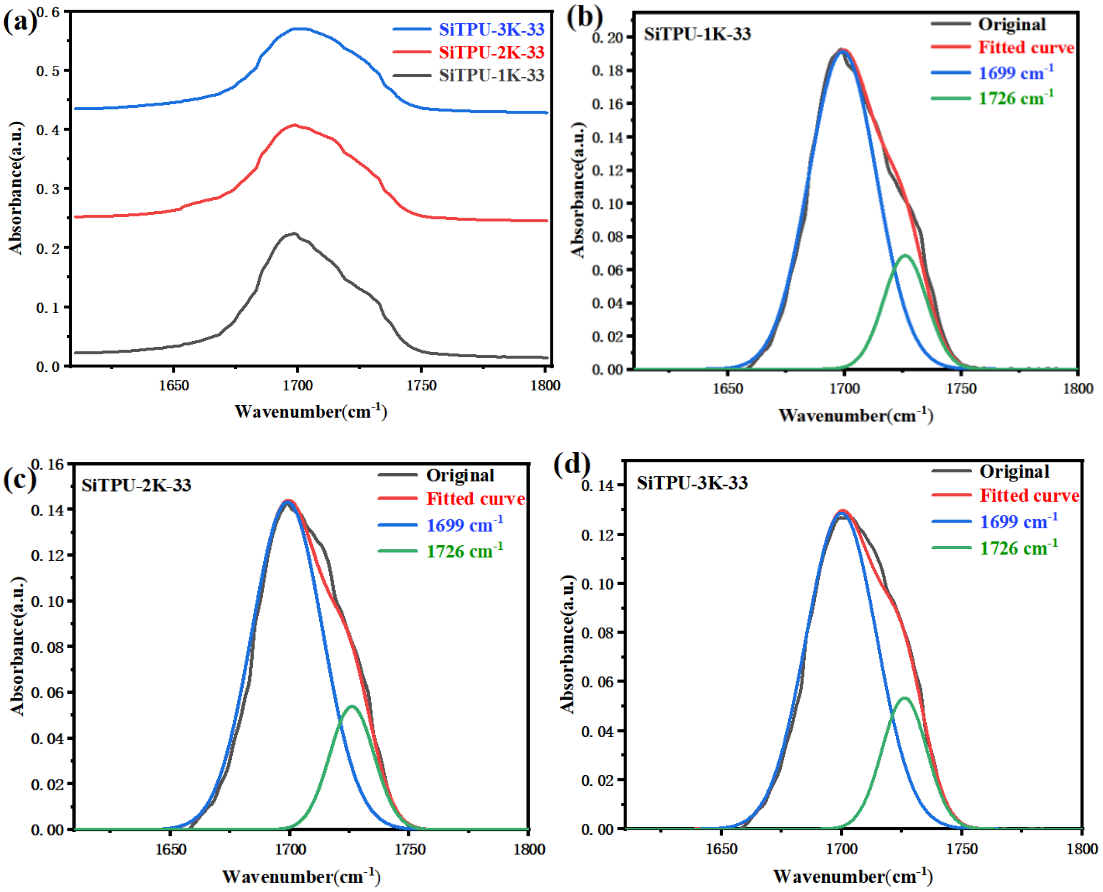

Fig S2. (a) FTIR spectra of Si-TPUs in the carbonyl regions from 1610 to 1800 cm^{-1}. Fitted Curve of the carbonyl groups of SiTPU-1K-33(b), SiTPU-2K-33 (c), SiTPU-3K-33 (d).

Table S1. Molecular weights and molecular weight distribution indices of Si-TPUs.

Sample	M_n (10^4)	M_w (10^4)	Đ
SiTPU-1K-33	5.1	11.5	2.2
SiTPU-2K-19	2.6	4.6	1.7
SiTPU-2K-23	2.6	5.0	1.9
SiTPU-2K-28	2.6	5.1	1.9
SiTPU-2K-33	2.8	5.4	1.9
SiTPU-2K-39	2.5	4.8	1.9
SiTPU-3K-33	3.6	7.8	2.1

Table S2. Periodic size (D) of the Si-TPUs.

Sample	Q (nm^{-1})	D (nm)
SiTPU-1K-33	0.1169	53.7
SiTPU-2K-19	0.1058	59.4
SiTPU-2K-23	0.0925	67.9
SiTPU-2K-28	0.0813	77.3
SiTPU-2K-33	0.0751	83.7
SiTPU-2K-39	0.0647	97.1
SiTPU-3K-33	0.0527	119.2

Table S3. Summary of the mechanical performance values of Si-TPUs.

Sample	Tensile stress （MPa）	Elongation at break （%）	Stress at 100% elongation （MPa）	Stress at 300% elongation （MPa）	Hardness （Shore A）	Toughness （MJ M^{-3}）
SiTPU-1K-33	17.0±0.3	776±17	3.6	6.7	74	65.4
SiTPU-2K-19	0.1±0.1	724±24	0.1	0.1	28	0.9
SiTPU-2K-23	1.7±0.3	510±17	1.3	1.7	51	7.9
SiTPU-2K-28	6.0±0.2	431±19	4.2	5.3	71	20.5
SiTPU-2K-33	11.8±0.4	425±16	6.8	9.6	78	35.8
SiTPU-2K-39	21.5±0.5	388±13	10.8	18.5	85	55.6
SiTPU-3K-33	11.8±0.4	210±15	9.5	-	81	19.1

Table S4. The mechanical performance values of Si-TPUs after multiple recycles.

Sample	Tensile stress (MPa)	Elongation at break (%)	Stress at 100% elongation (MPa)	Stress at 300% elongation (MPa)	Hardness (Shore A)	Toughness (MJ M^{-3})
SiTPU-2K-39	21.5±0.5	388±13	10.8	18.5	85	55.6
1st	19.3±0.3	386±14	9.7	16.6	85	49.8
2nd	18.5±0.2	380±12	9.4	16.1	85	46.9
3rd	17.4±0.5	367±13	8.9	15.3	85	42.6

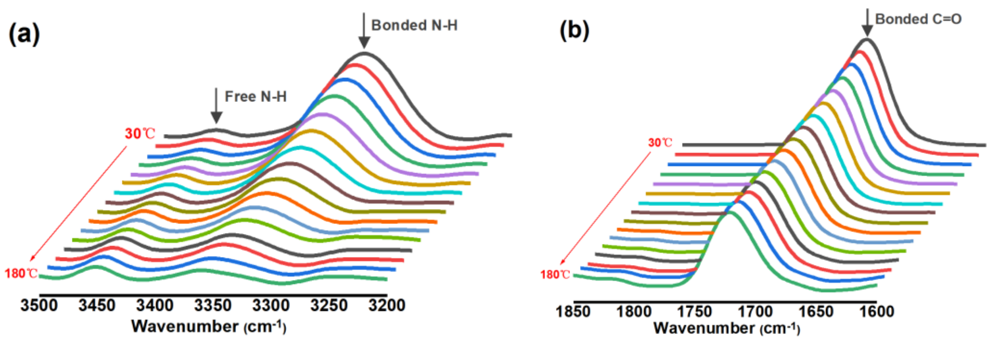

Fig S3. FTIR spectra of (a)N–H and (b)C=O from SiTPU-2K-39.
